# Advances in Targeted Therapy for Non-Small-Cell Lung Cancer: Current Progress and Future Directions

**DOI:** 10.3390/ijms262311517

**Published:** 2025-11-27

**Authors:** Supriya Peshin, Ehab Takrori, Joseph H. Yazji, Johum Haque, Adit Dharia, Mohammad Sajid Mithani, Fnu Anum, Ummul Asfeen, Jill Kristen Couch, Mabe Donovan, Sakshi Singal

**Affiliations:** 1 Department of Internal Medicine, Norton Community Hospital, Norton, VA 24273, USA; johum.haque@balladhealth.org (J.H.); fnu.anum2@balladhealth.org (F.A.); jcouch_nch@yahoo.com (J.K.C.); 2 College of Medicine, Alfaisal University, Riyadh 11533, Saudi Arabia; etakrori@gmail.com (E.T.); mohammedsajid2014@gmail.com (M.S.M.); 3 HCA Florida Oak Hill Hospital, Brooksville, FL 34613, USA; drjyazji@gmail.com (J.H.Y.); aditdha@gmail.com (A.D.); 4 MD Anderson Cancer Center, 1515 Holcombe Blvd, Houston, TX 77030, USA; uzasfeen@mdanderson.org; 5 Department of Pulmonary Critical Care, Norton Community Hospital, Norton, VA 24273, USA; donovan.mabe@balladhealth.org; 6 Department of Hematology and Oncology, East Tennessee State University, Johnson City, TN 37614, USA

**Keywords:** targeted therapies, non-small cell lung cancer, NSCLC, EGFR, ALK, KRAS, MET, HER2, RET, TROP-2, BRAF V600E, NTRK

## Abstract

The advent of targeted therapies has significantly transformed the management of non-small-cell lung cancer (NSCLC), improving survival across all disease stages. Discoveries of both common and rare oncogenic drivers are advancing rapidly, posing a challenge for clinicians and researchers to remain up to date in this dynamic field. This review highlights the evolving landscape of therapeutic strategies for actionable mutations in lung cancer, with particular attention given to the latest developments in KRAS-targeted treatments including non-G12C mutations, pan-RAS inhibitors, and agents targeting RAS-GTP. We also examine the existing standards of care for NSCLC harboring EGFR and ALK alterations, as well as emerging therapies poised for clinical use. Additional discussion includes advancements in therapies directed at MET, HER2, RET, ROS1, and FGFR alterations—each representing promising targets in NSCLC. This review concludes by exploring the growing evidence surrounding TROP-2 as a novel therapeutic target, especially relevant in cases where previous targeted treatments have failed.

## 1. Introduction

Lung cancer remains one of the most frequently diagnosed malignancies worldwide and is the leading cause of cancer-related mortality [1]. Non-small-cell lung cancer (NSCLC), which accounts for approximately 85% of cases, was historically managed with cytotoxic chemotherapy. Over the past two decades, however, advances in molecular diagnostics and targeted therapies have fundamentally reshaped its treatment landscape.

The integration of next-generation sequencing (NGS) into routine clinical practice has significantly expanded our understanding of NSCLC’s molecular heterogeneity. In particular, liquid biopsies—the analysis of circulating tumor DNA (ctDNA) in blood—now complement tissue testing by detecting oncogenic drivers and resistance mutations. These techniques have enabled the discovery of numerous oncogenic drivers and the development of targeted therapies that have improved outcomes for molecularly selected patients. Landmark discoveries, such as the epidermal growth factor receptor (EGFR) and rearrangements involving Anaplastic Lymphoma Kinase (ALK), ROS proto-oncogene 1 (ROS1), and BRAF, have led to the approval of multiple Tyrosine Kinase Inhibitors (TKIs) and redefined first-line treatment options [2].

Between 2003 and 2020, targeted therapies evolved from first-generation agents to more selective compounds, leading to the development of third-generation EGFR TKI’s (e.g., osimertinib) and next-generation ALK/ROS1 inhibitors (e.g., lorlatinib) [3,4,5]. Although these breakthroughs represent major progress, their clinical utility is limited to patients whose tumors harbor specific driver mutations. Consequently, research efforts have increasingly focused on expanding the spectrum of targetable alterations, leading to multiple recent U.S. Food and Drug Administration (FDA) approvals and an active pipeline of investigational agents. The aim of this review is to synthesize recent advances in targeted therapy for NSCLC, focusing on the clinical progress, molecular mechanisms, and current as well as future direction of the therapies directed at key oncogenic divers.

This review provides a comprehensive overview of the evolving therapeutic landscape of NSCLC, with an emphasis on emerging targeted treatments for established oncogenic drivers. We summarize current molecular targets and therapeutic approaches supported by recent clinical evidence and practice guidelines, and highlight investigational agents presented at major oncology conferences, including ASCO, AACR, and WCLC. While several recent reviews have summarized targeted therapies for NSCLC, the present manuscript integrates 2024–2025 clinical and regulatory updates, links discovery methods (e.g., crystallography, structure-based modeling, RNA sequencing) directly to drug design and resistance biology, and discusses current trial statuses, including terminated studies. We also include targets such as TROP-2 and FGFR and emphasize mechanism-anchored narratives at the mutation level, distinguishing this work from earlier synthesis [6,7,8].

**Figure 1:**
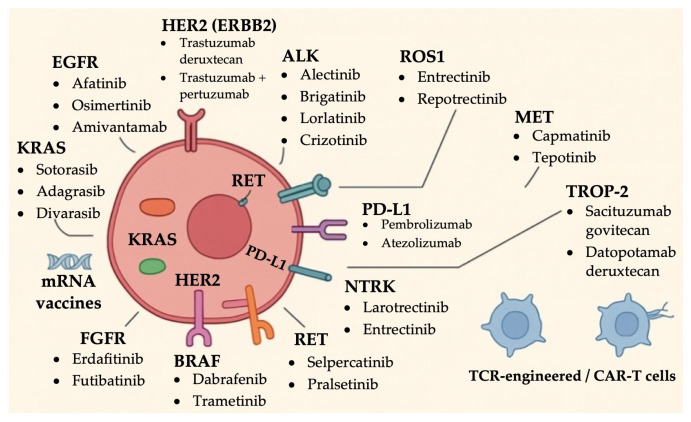
Summary of established targeted therapies in NSCLC based on actionable driver mutations.

Key molecular targets discussed and summarized in Table 1 include Kirsten rat sarcoma viral oncogene homolog (KRAS), EGFR, Human Epidermal Growth Factor Receptor (HER2), Mesenchymal-Epithelial Transition (MET), ALK, rearranged during transfection (RET), V-Raf Murine Sarcoma Viral Oncogene Homolog B (BRAF V600E), Neurotrophic Tyrosine Receptor Kinase (NTRK), ROS1, Trophoblast Cell Surface Antigen 2 (TROP-2), and Fibroblast Growth Factor receptor (FGFR) (Figure 1). Notably, this review does not address the Rapidly Accelerated Fibrosarcoma (RAF) and Mitogen-Activated Protein Kinase (MEK) signaling pathways, which are considered outside the scope of our discussion.

## 2. Discussion

### 2.1. Molecular Testing and Diagnostic Strategies

Current consensus guidelines from the College of American Pathologists (CAP), the International Association for the Study of Lung Cancer (IASLC), and the Association of Molecular Pathology (AMP) recommend front line comprehensive genomic profiling by NGS on tumor tissues for all patients with advanced NSCLC. This single assay approach simultaneously interrogates EGFR, ALK, ROS1, BRAF, KRAS, MET exon 14 skipping, RET, NTRK, HER2 and other emerging targets, conserving tissue and avoiding the delays of sequential single gene tests [21]. In routine practice, hybrid capture DNA/RNA panels such as FoundationOne CDx, Oncomine Comprehensive Assay and TruSight Oncology return results within 5–10 days and identify single-nucleotide variants, small insertion/deletions, copy number changes, and gene fusions in one run, thereby enabling rapid therapy selection [22].

When tissue is insufficient or a faster answer is required, liquid biopsy (ctDNA NGS) offers a minimally invasive alternative with high concordance for actionable driver mutations and a median turnaround of 7 days with a range of 5–22 days [23]. Studies now endorse liquid biopsy as an acceptable first line test when tissues cannot be obtained or as a complementary tool for real time disease monitoring [24]. Integrating tissue and blood-based platforms can lift the actionable alteration yield to higher ratios while shortening time to treatment initiation, an increasingly important metric in modern NSCLC care [25,26].

Beyond NGS and ctDNA profiling, recent advances in structural biology and spatial multi-omics have accelerated the development of targeted therapies. X-ray crystallography and structure-based modeling enabled inhibitor classes for mutated KRAS and resistant RET variants, while RNA-sequencing/fusion calling derived therapy for rare fusions (e.g., FGFR3-TACC3). Emerging spatial omics further contextualizes resistance niches and heterogeneity, thereby providing a rational combination [8].

### 2.2. EGFR

EGFR is a transmembrane receptor tyrosine kinase that regulates cell proliferation, survival, and differentiation. Overexpression of EGFR in various malignancies was first observed in the late 1980s and was subsequently recognized as a key oncogenic driver in NSCLC [27]. Identification of EGFR as a viable therapeutic target fundamentally transformed the lung cancer treatment landscape [27].

First-generation reversible EGFR TKIs, such as gefitinib and erlotinib, marked the beginning of targeted therapy for EGFR-mutant NSCLC. These agents bind the ATP-binding site of the EGFR kinase domain and, in pivotal trials, produced superior progression-free survival (PFS) and response rates compared to chemotherapy. Tumors harboring exon 19 deletions (Ex19del) and the exon 21 L858R point mutation are especially sensitive; however, most patients developed resistance within approximately one year of treatment, commonly due to the emergence of the T790M gatekeeper mutation in exon 20 [28].

Second-generation EGFR TKIs, including afatinib and dacomitinib, were developed as irreversible inhibitors to address this resistance. These agents also target other members of the ErbB receptor family. Although they offered broader inhibitory activity, their clinical utility was limited by higher toxicity and insufficient efficacy against T790M-positive tumors [29].

Third-generation EGFR TKIs, such as osimertinib, represented a significant advancement. Osimertinib was specifically engineered to inhibit both sensitizing EGFR mutations and the T790M resistance mutation while sparing wild-type EGFR to reduce toxicity. This agent has become the preferred first-line therapy for EGFR-mutated NSCLC, demonstrating superior outcomes in both overall survival (OS) and PFS compared to earlier-generation TKIs [30,31].

More recently, the FLAURA-2 phase III international trial (NCT04035486) evaluated the efficacy of osimertinib in combination with pemetrexed and platinum-based chemotherapy in patients with advanced NSCLC harboring Ex19del or L858R mutations. The combination significantly extended PFS to 25.5 months compared to 16.7 months with osimertinib alone (HR 0.62; 95% CI, 0.49–0.79; *p* < 0.001) [32]. Notably, in patients with baseline central nervous system (CNS) involvement, PFS was 24.9 months with the combination versus 13.8 months with monotherapy (HR 0.47; 95% CI, 0.33–0.66) [33].

Amivantamab, a bispecific EGFR/MET antibody, was initially approved for NSCLC patients with exon 20 insertion mutations (Exon20ins), which are typically resistant to traditional EGFR TKIs [34,35]. The MARIPOSA phase III trial (NCT04487080) compared amivantamab and Lazertinib with Osimertinib in treatment-naïve Ex19del/L858R NSCLC and achieved a median PFS of 23.7 months, surpassing osimertinib’s 16.6 months (HR 0.70; 95% CI, 0.58–0.85; *p* < 0.001), along with a longer duration of response (DOR) (25.8 months versus 16.8 months) (95% CI: 20.1–NE and 14.8–18.5, respectively). These gains were accompanied by a higher incidence of grade ≥ 3 adverse events (AEs), including paronychia, rash, infusion-related reactions, and venous thromboembolic events (VTEs) [36]. Given the increased VTE risk in the initial four months of treatment, prophylactic anticoagulation during this initial period is now recommended by the FDA [37,38]. A subcutaneous formulation reduced infusion-related reactions from 63% to 14% in the PALOMA-3 trial (NCT05388669) while preserving efficacy [39].

The MARIPOSA-2 trial (NCT04988295) randomized patients with NSCLC who had progressed on Osimertinib to one of three regimens: amivantamab + lazertinib with chemotherapy, amivantamab with chemotherapy, or chemotherapy alone. Both amivantamab-containing arms demonstrated improved PFS—8.3 and 6.3 months, respectively, versus 4.2 months with chemotherapy alone (HRs: 0.44 and 0.48, both *p* < 0.001) [40]. While higher-grade AEs were more common in the triplet arm, including neutropenia, thrombocytopenia, rash, and VTEs, the improved efficacy has made amivantamab–chemotherapy a preferred option following osimertinib failure.

Similarly, the PAPILLON trial (NCT04538664) investigated the use of amivantamab in combination with chemotherapy, extending the PFS to 11.4 months compared to 6.7 months with chemotherapy alone, translating to a 40% reduction in the risk of disease progression or death (HR 0.395; 95% CI, 0.30–0.53; *p* < 0.0001) [41].

Clinical decision making must be individualized. Factors such as age, performance status, presence of brain metastases, ctDNA levels, co-mutation profiles (e.g., TP53, RB1), and eligibility for chemotherapy often favor combination regimens over Osimertinib monotherapy [42,43].

A recent press release reported that the combination of amivantamab and lazertinib may extend median OS by more than one year compared to osimertinib alone, a finding expected to influence future treatment paradigms once full data become available [44]. Resistance analyses from the MARIPOSA trial revealed lower rates of MET amplification and secondary EGFR mutations with combination therapy, likely due to dual-pathway inhibition [45].

Sunvozertinib, an irreversible exon20ins-active TKI, achieved a 54% ORR and 91% disease control rate (DCR) in heavily pretreated patients in the phase II WU-KONG1 trial (NCT03974022). The confirmatory WU-KONG28 trial (NCT05668988) is currently underway to further assess its safety and efficacy [35,46].

Furmonertinib, another third-generation EGFR TKI with strong CNS penetration, was initially approved in China for NSCLC patients with EGFR T790M mutations. The FURLONG phase III trial (NCT03787992) demonstrated improved PFS with furmonertinib compared to gefitinib in the first line setting for EGFR-mutated NSCLC [47]. In a phase I study of patients with exon20ins-mutant NSCLC, furmonertinib 240 mg led to a 69% ORR and a median PFS of 10 months in treatment-naïve individuals. For pretreated patients, median PFS was 7.0 months and 5.8 months with the 240 mg and 160 mg doses, respectively [48]. Furmonertinib is currently being studied in the FURVENT/FURMO-004 global phase III trial (NCT05607550) as a potential first-line option for this patient group. The FDA has granted it breakthrough therapy designation for the treatment of untreated advanced NSCLC with EGFR exon20ins mutations.

Despite notable progress in EGFR-targeted therapies, treatment options following disease progression remain limited. HER3 is often upregulated in EGFR-mutant NSCLC and is implicated in resistance to EGFR TKIs [49], making it an appealing therapeutic target. Patritumab deruxtecan (HER3-DXd), an antibody–drug conjugate (ADC), links a HER3-directed monoclonal antibody with a topoisomerase I inhibitor payload, facilitating direct delivery of cytotoxic therapy to HER3-overexpressing cells [50]. Early clinical studies have shown encouraging activity, including improved PFS in patients with advanced NSCLC who had progressed on EGFR TKIs [51]. In the HERTHENA-Lung01 phase II trial (NCT04619004), HER3-DXd yielded an ORR of 29.8% (95% CI, 23.9–36.2), a median PFS of 5.5 months (95% CI, 5.1–5.9), and median OS of 11.9 months (95% CI, 11.2–13.1), with a manageable toxicity profile and low discontinuation rates [52]. Further evidence on efficacy is expected from the ongoing phase III HERTHENA-Lung02 trial (NCT05338970). A pipeline of additional EGFR-targeted agents including bispecific and trispecific antibodies, ADCs, and cellular therapies is currently in development [53].

**Figure 2:**
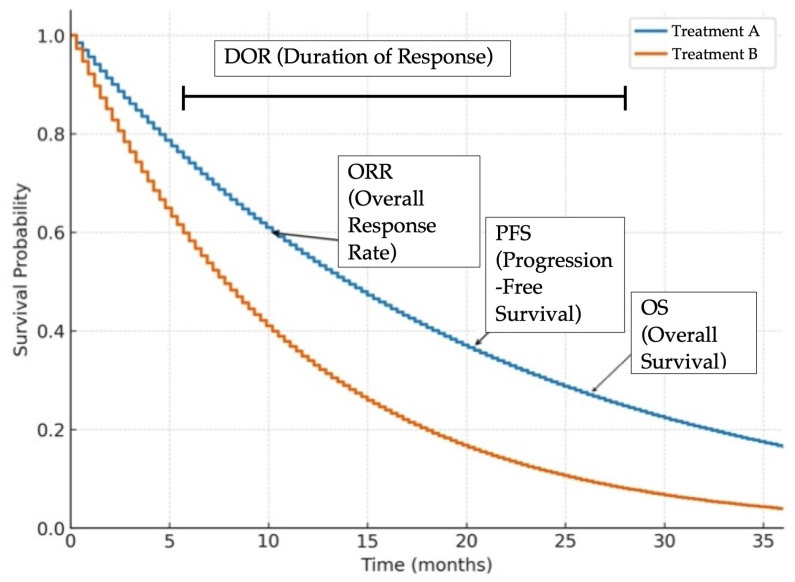
Representative Kaplan–Meier survival curves illustrating common clinical trial endpoints in oncology. Overall survival (OS) is the time from treatment initiation until death from any cause. Progression free survival (PFS) is the time from treatment initiation until disease progression or death. Overall response rate (ORR) is the proportion of patients achieving a predefined amount of tumor shrinkage according to RECIST criteria, regardless of duration. Duration of response (DOR) is the time from initial tumor response to disease progression or death. These curves are for illustrative purposes only and do not represent actual patient data.

In addition to the well-characterized EGFR mutations, approximately 10–15% of cases involve uncommon variants [9,54]. Among these, G719X, L861Q, S768I, E709X, E709-T710delinsD, and L747X are most frequently encountered [55,56,57]. Based on structural features, these mutations have been categorized into a group known as P-loop and alpha-helix compressing mutations (PACC), according to the MD Anderson Cancer Center EGFR classification system [58]. These mutations typically show lower sensitivity to EGFR TKIs, although some such G719X respond better especially to second-generation agents [59]. In a retrospective analysis, afatinib was associated with favorable responses in patients with these rare mutations [60]. The ACHILLES/TORG1834 phase III trial (jRCTs031180175) further confirmed improved PFS with afatinib compared to platinum–pemetrexed chemotherapy (10.6 vs. 5.7 months; HR 0.42; 95% CI, 0.26–0.694; *p* = 0.0007) [61]. However, the lack of CNS activity remains a clinical gap in treating PACC-mutant NSCLC. A novel investigational agent, ORIC-114 (NCT05315700), is a brain penetrant and highly selective EGFR/HER2 inhibitor based on promising preclinical data from structure-based drug design and modeling studies to optimize blood–brain barrier penetration and selectivity for atypical EGFR mutations. It is currently being assessed in a phase I/II trial for its potential activity against exon20ins and other atypical EGFR mutations, based on promising preclinical data [62]. Emphasizing structure guided CNS penetration as a design goal distinguishes this generation of EGFR/HER2 inhibitors and may explain intracranial activity patterns. Figure 2 provides an illustrative overview of the clinical trial endpoints—OS, PFS, ORR, DOR—for clarity of interpretation. Table 2 provides a summary of clinical trials targeting EGFR-mutant tumors, explaining their phases, ORR, and median PFS. All trials in Table 2, Table 3 and Table 4 are status verified through August 2025; terminated studies are explicitly labeled to reflect real time development trajectories.

### 2.3. ALK

ALK rearrangements were initially identified in large-cell lymphomas, but the discovery of the EML4-ALK fusion in NSCLC in 2007 established ALK as a key oncogenic driver in this disease [92,93]. ALK fusions are present in approximately 4–7% of NSCLC cases [94]. The gene encodes a receptor tyrosine kinase that is normally expressed during embryonic development of the nervous system but remains largely inactive in adult tissues, making it an ideal therapeutic target when aberrantly activated in cancer.

In ALK-rearranged NSCLC, fusion proteins such as EML4-ALK promote continuous activation of downstream signaling pathways including MAPK, PI3K-AKT-mTOR, and JAK-STAT leading to uncontrolled cell proliferation. Crizotinib was the first ALK inhibitor approved for treatment, but its limitations included poor penetration into the CNS and the emergence of resistance mutations [95].

Second-generation ALK TKIs—alectinib, brigatinib, and ceritinib—became standard first-line therapies due to their improved efficacy and CNS activity [96,97,98]. Lorlatinib, a third-generation ALK inhibitor initially approved for use following progression on second-generation TKIs, demonstrated efficacy against the G1202R resistance mutation [99]. In the phase III CROWN trial, lorlatinib significantly prolonged PFS, which was not reached at the time of analysis, compared with 9.3 months for crizotinib (HR 0.28; 95% CI, 0.19–0.41; *p* < 0.001) [100]. The five-year PFS rate was 60% for lorlatinib compared to 8% for crizotinib [101].

Certain high-risk factors such as EML4-ALK variant 3, TP53 co-mutations, brain metastases, and elevated ctDNA are linked to poorer outcomes with earlier-generation ALK inhibitors [102]. In ALK-positive NSCLC, elevated ctDNA levels in particular have been associated with reduced PFS on first and second generation ALK TKIs. Notably, lorlatinib maintained its efficacy in these high-risk subgroups, reinforcing its role as a preferred frontline agent [101]. Despite its potency, lorlatinib has a distinct AEs profile, including hyperlipidemia (cholesterol: 72%, triglycerides: 66%), edema (57%), weight gain (44%), and cognitive or mood disturbances (49%) [101]. No direct head-to-head comparisons among ALK TKIs exist, but network meta-analyses suggest lorlatinib has the highest efficacy, whereas low dose alectinib may offer better tolerability, particularly in patients with CNS involvement [10].

Lorlatinib’s off-target inhibition of TRKA/B/C receptors is believed to contribute to its cognitive side effects and weight gain [103]. To address these limitations, newer generation ALK inhibitors are in development. Deulorlatinib, a deuterated analog of lorlatinib, has demonstrated high intracranial activity and an ORR of 87.9% in TKI-naïve patients and 38.1% in patients previously treated with second-generation TKIs [104]. NVL-655, a fourth-generation ALK inhibitor designed to spare TRK inhibition, retains potent activity against resistant mutations, including compound mutations like G1202R/L1196M. In a phase I/II trial, the ORR was 37% among patients previously treated with three ALK TKIs, including lorlatinib, and 53% among those who had not received lorlatinib [64,66].

### 2.4. ROS1

The ROS1 gene encodes a receptor tyrosine kinase typically active during embryogenesis and largely silent in adult tissues [11]. Oncogenic ROS1 rearrangements caused by chromosomal translocations result in constitutively active fusion proteins that drive cancer progression via MAPK, PI3K, and JAK/STAT signaling pathways [105]. These rearrangements are observed in approximately 1–2% of NSCLC cases, predominantly in younger, non-smoker patients with adenocarcinoma histology [105,106].

Crizotinib was the first approved therapy for ROS1-rearranged NSCLC and demonstrated substantial clinical activity, regardless of the fusion partner [70]. It has limited CNS penetration and developed rapid emergence of resistance mutations, however, frequently leading to relapse—particularly in patients with brain metastasis [72,107,108].

Newer ROS1-targeted TKIs such as ceritinib, entrectinib, and lorlatinib have improved CNS efficacy and are now integral to treatment strategies [74]. Entrectinib received regulatory approval following pooled analyses from the AL-KA-372-001, STARTRK-1, and STARTRK-2 trials, which showed an ORR of 68% (95% CI, 60.2–74.8) and a median DOR of 20.5 months. It had a manageable safety profile, with grade ≥ 3 AEs in 56.4% of patients with weight gain being the most common adverse event [68].

Repotrectinib, a next-generation ROS1/ALK/TRK inhibitor, was approved based on the TRIDENT-1 phase II trial. In TKI-naïve patients, repotrectinib achieved an ORR of 79% (95% CI, 68–88) and a median PFS of 34.1 months. In previously treated patients, ORR was 38% (95% CI, 25–52) with a PFS of 9.0 months [109]. Notably, repotrectinib retains activity against resistance mutations such as the solvent-front G2032R variant [110].

NTRK gene fusions are rare, but potentially actionable oncogenic drivers in NSCLC, typically presenting in <1% of patients—often younger, non-smokers with adenocarcinoma histology. Larotrectinib is an FDA-approved selective TRK inhibitor for NTRK fusion-positive solid tumors regardless of histology, thus making it a tumor-agnostic therapy. The case report (PMC10601816) presents the first documented complete response in an NSCLC patient with an NTRK2 fusion treated with larotrectinib [12].

Taletrectinib, a CNS-penetrant ROS1/TRK inhibitor, has received FDA Breakthrough Therapy Designation for advanced ROS1-positive NSCLC. Combined analysis of the TRUST-I (NCT04395677) and TRUST-II (NCT04919811) trials reported an ORR of 92% (95% CI, 85.4–95.7) in TKI-naïve patients, with PFS not yet reached. In pretreated patients, ORR was 54%, with a median PFS of 9.6 months [76,80]. The treatment was well tolerated, with the most common AEs being elevated liver enzymes, dizziness, and diarrhea, and only 6% of patients discontinued therapy due to toxicity.

Given the compelling intracranial efficacy, entrectinib and repotrectinib are often preferred first-line options in patients with ROS1-rearranged NSCLC and brain metastases. Ongoing strategies aim to enhance efficacy and delay resistance by combining ROS1 TKIs with other agents. For instance, EGFR and MET activation are known resistance pathways [82,111], and combinations with agents like amivantamab are currently under investigation in patients with ALK, ROS1, and RET fusions (NCT05845671) [82,111].

### 2.5. BRAF V600E

BRAF V600E mutations are less common than EGFR and ALK alterations; however, these represent a distinct subset of NSCLC with an FDA-approved treatment strategy. The combination of dabrafenib and trametinib has shown durable responses in clinical trials and is supported by real-world data. A recent Italian retrospective analysis explored this combination in 42 patients treated at a single institute across six years. The median PFS was 19.9 months with first-line treatment versus 13.1 months with later lines. The median OS across all lines of therapy was 29.9 months with an ORR of 73.8%. Notably, the therapy was well tolerated even in elderly (median age 72), and no patients had to stop treatment due to side effects. These findings validate the tolerability and durability of BRAF/MEK inhibition in real world settings, especially when initiated early [13].

Similar oncogenic drivers, including mutations in KRAS, BRAF, and HER2 (ERBB2), have been identified in intrahepatic cholangiocarcinoma (iCCA). BRAF V600E mutations occurred in 3–7% of iCCA cases and were associated with poorer prognosis, while HER2 amplification or overexpression occurred in 5–20% of cholangiocarcinoma cases. KRAS G12C mutations occurred in a limited number of patients and had promising results when targeted with adagrasib. Therapeutics targeting BRAF V600E mutations (e.g., dabrafenib + trametinib), HER2 amplifications or overexpression (e.g., trastuzumab-deruxtecan, trastuzumab + pertuzumab), and KRAS G12C mutations (e.g., adagrasib) are either in clinical use or under active investigation [112].

### 2.6. KRAS

KRAS, a small GTPase encoded on chromosome 12p12.1, plays a pivotal role in transmitting intracellular signals. In its active, GTP-bound form, KRAS initiates downstream signaling via the RAS/RAF/MEK/ERK (MAPK), PI3K/AKT/mTOR, and RalGDS/Ral pathways, ultimately promoting cell growth, survival, and metastasis [85,113,114]. Activation is triggered when extracellular signals engage receptor tyrosine kinases such as EGFR, ALK, MET, HER2, RET, FGFR, and NTRK, leading to recruitment of adaptor proteins like growth factor receptor-bound protein 2 (GRB2) and guanine nucleotide exchange factors (GEFs) such as Son of Sevenless homolog 1 (SOS1) [115]. GEFs catalyze GDP release from KRAS, enabling cytosolic GTP binding and switching KRAS to its “ON” state. Scaffolding protein SHP2 (Src homology region 2-containing protein tyrosine phosphatase 2) (PTPN11) stabilizes RTK-GRB2-SOS1 signaling, facilitating GDP to GTP state switching [116]. Signal termination requires intrinsic KRAS GTPase activity, strongly accelerated by GTPase-activating proteins (GAPs) such as NF1 and RASA1, which hydrolyze GTP back to GDP, returning KRAS to the inactive state [117].

Oncogenic mutations, particularly at codons 12, 13, and 61, impair GTP hydrolysis and disrupt GAP binding, resulting in constitutive KRAS activation and unregulated downstream signaling. Approximately 30% of lung adenocarcinomas harbor KRAS mutations [89,91,118], with the G12C variant being the most prevalent, comprising about 40% of KRAS-altered NSCLC cases [119]. Historically, KRAS was deemed “undruggable” due to its high affinity for GTP and lack of a suitable binding pocket [120]. However, the development of covalent inhibitors capable of binding irreversibly to the cysteine residue unique to KRAS G12C marked a breakthrough in cancer therapeutics. These inhibitors trap KRAS in its GDP-bound inactive form, suppressing downstream signaling [120]. Additional strategies now target other steps in the KRAS cycle: SHP2 inhibitors block upstream RTK → SOS1 activation [116]; G12D and pan-KRAS inhibitors, as well as RAS-ON inhibitors disrupt active KRAS signaling [121,122,123]; KRAS degraders promote proteasomal elimination [121]; and downstream BRAF/MEK/ERK inhibitors block MAPK output irrespective of KRAS mutation status [85,113,121].

Sotorasib was the first KRAS G12C inhibitor tested clinically in the phase I/II CodeBreaK 100 trial (NCT03600883), which enrolled patients with advanced solid tumors, including NSCLC, who had previously received standard therapies. In NSCLC patients, the ORR was 37% (95% CI: 28.6–46.2), with some responses lasting over 12 months. The median PFS was 6.8 months (95% CI: 5.1–8.2), and the median OS was 12.5 months (95% CI: 10.0–NE) [121]. The most frequent AEs included nausea, fatigue, arthralgia, and transient elevations in liver enzymes (ALT and AST).

Adagrasib, another KRAS G12C inhibitor, showed comparable activity in the KRYSTAL-1 phase II trial (NCT03785249). Among 112 previously treated NSCLC patients, the ORR was 42.9%, with a median DOR of 8.5 months (95% CI: 6.2–13.8), a PFS of 6.5 months (95% CI: 4.7–8.4), and an OS of 12.6 months (95% CI: 9.2–19.2) [122,123]. Notably, adagrasib demonstrated intracranial efficacy in patients with untreated brain metastases, achieving a 33.3% intracranial ORR (95% CI: 20.3–66.5) [87]. Common adverse effects included nausea, diarrhea, fatigue, musculoskeletal pain, liver toxicity, and renal function abnormalities [123].

These results supported the launch of phase III trials. In CodeBreaK 200 trial (NCT04303780), sotorasib was compared to docetaxel in pretreated KRAS G12C-mutant NSCLC. Sotorasib achieved a higher ORR (28% vs. 13%) and significantly improved PFS (5.6 vs. 4.5 months; HR: 0.66; 95% CI: 0.51–0.86; *p* = 0.0017). However, OS was not significantly different (10.6 vs. 11.3 months; HR: 1.01; 95% CI: 0.77–1.33) [124]. Similarly, the KRYSTAL-12 trial (NCT04685135) demonstrated superior efficacy of adagrasib over docetaxel, with a PFS of 5.5 vs. 3.8 months (HR: 0.58; 95% CI: 0.45–0.76; *p* < 0.0001) and ORR of 32% vs. 9% [125].

Newer more potent and selective agents are now entering clinical development. Divarasib, a highly selective KRAS G12C inhibitor, has shown promising efficacy: in a phase I trial (NCT04449874) involving NSCLC patients, the ORR was 53% (95% CI: 39.9–66.7), and AEs were predominantly mild gastrointestinal AEs [126,127]. A phase III trial (Krascendo 1, NCT06497556) is underway to compare divarasib with sotorasib or adagrasib.

Opunarasib, another novel inhibitor targeting the switch II pocket (S-IIP), irreversibly inactivates KRAS G12C. Preclinical studies have shown mutant-selective, dose-dependent inhibition of tumor growth [128]. In early-phase clinical trials, opunarasib yielded a 42% ORR in NSCLC patients [129]. Ongoing studies include KontRASt-02 (NCT05132075), a phase III comparison with docetaxel, and KontRASt-03 (NCT05358249), evaluating combinations with agents such as tislelizumab, trametinib, ribociclib, and cetuximab.

Additional G12C inhibitors olomorasib, garsorasib, and others are currently in various stages of clinical evaluation [130,131]. Resistance to KRAS G12C inhibitors can arise through feedback activation of the RAS-MAPK pathway, receptor tyrosine kinase signaling (e.g., MET amplification), or alternative mechanisms like IGF pathway activation and loss of E-cadherin [132,133,134]. Studies have also identified synthetic lethal vulnerabilities involving kinases, tRNA-modifying enzymes, and the YAP/TAZ/TEAD axis [135,136].

These challenges have led to the development of next-generation inhibitors and KRAS degraders. Encouraged by the success of G12C inhibitors, efforts are now focused on targeting other KRAS mutations. After G12C, the most common KRAS alterations in NSCLC are G12V (~19%) and G12D (~15%) [137,138]. These mutations lack a reactive cysteine, necessitating novel, non-covalent targeting strategies.

MRTX-1133 is a selective non-covalent KRAS G12D inhibitor that binds both active and inactive conformations. Preclinical models have demonstrated robust, dose-dependent tumor regression [139,140], which prompted a phase I/II trial (NCT05737706) that was later terminated. HRS-4642 is another G12D inhibitor with early-phase clinical data showing tumor shrinkage in 33% of heavily pretreated patients, including those with colorectal cancer, and an ORR of 10% in NSCLC patients (NCT05533463) [141]. Proteasome inhibitors such as carfilzomib have shown potential to enhance HRS-4642 efficacy [142].

Another G12D-selective agent, LY3962673, is now being evaluated in the MOONRAY-01 phase 1 trial (NCT06586515). It has demonstrated promising preclinical activity in xenograft models [143]. Other agents in preclinical or early clinical development include QTX3046 (NCT06428500) and INCB161734 (NCT06179160), which are part of a growing arsenal of therapies aimed at overcoming the limitations of current KRAS-targeted treatments [144,145]. Recent studies also suggest that structural differences between KRAS G12D and other isoforms, including their preferences for GTP- vs. GDP-bound states, may influence both drug design and response. Leveraging these differences in switch regions and salt bridge formation is a key area of future development [14]. 

The KRAS G12D mutant protein predominantly exists in its active, GTP-bound “ON” state, which contributes to persistent signaling and tumorigenesis [146]. This biochemical property has necessitated the development of inhibitors that specifically target KRAS in its active conformation, known as RAS-ON inhibitors. However, designing such inhibitors has been particularly challenging due to KRAS′s high affinity for GTP and the absence of deep binding pockets. To circumvent these obstacles, researchers developed a tricomplex RAS-ON inhibition strategy—revealed through crystallographic studies and structure-based modeling of cyclophilin A KRAS interface—where the chaperone protein that enables cooperative binding to the S-IIP of KRAS, facilitates covalent engagement with the aspartic acid residue unique to G12D. These method-resolved conformations (open P-loop in G13D; cyclophilin A stabilized RAS-ON complexes in G12D) map directly to inhibitor classes (allosteric GDP state binders vs. tricomplex RAS-ON agents), clarifying why drug susceptibility varies by allele and state [147,148].

RMC-9805 exemplifies this approach (NCT06040541). As a tricomplex KRAS G12D-ON inhibitor, it has demonstrated robust tumor regression in preclinical models of KRAS G12D-mutant cancers, including NSCLC. It is currently undergoing phase I clinical evaluation to assess its safety and therapeutic potential [147].

Another innovative direction involves targeted protein degradation. KRAS degraders, often designed as proteolysis-targeting chimeras (PROTACs), feature dual binding domains: one targets the mutant KRAS protein, while the other recruits an E3 ubiquitin ligase. This facilitates ubiquitination and subsequent proteasomal degradation of KRAS [149,150]. ASP3082 is a first-in-class KRAS G12D-targeted PROTAC that has shown promising, dose-dependent antitumor activity in preclinical KRAS G12D-mutant NSCLC models [151]. A first-in-human phase I study is currently ongoing (NCT05382559). Futuristically, refining biomarker selection, such as the detection of KRAS allele frequency, co-mutations in STK11, and patterns of immune evasion, may further guide patient satisfaction and combination therapy strategies for KRAS G12D-directed treatments [14]. 

In parallel, pan-KRAS inhibitors are being developed to inhibit a broad spectrum of KRAS mutations. These agents preferentially bind the inactive (GDP-bound) form of KRAS and block nucleotide exchange, thereby preventing activation. Targeted mutations include G12A/C/D/F/V/S, G13C/D, V14I, L19F, Q22K, D33E, Q61H, K117N, and A146V/T [152]. Most of these agents are in early-phase or preclinical development.

QTX3034 is a non-covalent pan-KRAS inhibitor that has shown high potency against KRAS G12D and moderate activity against G12V in preclinical models. It is currently being tested in early-phase trials for KRAS G12D-mutant tumors (NCT06227337) [153,154]. BI-3706674, another pan-KRAS agent, demonstrates selective inhibition of KRAS G12V and KRAS wild-type amplifications and is under phase I evaluation (NCT06056024) [155]. Similarly, JAB-23425 is an orally bioavailable pan-KRAS inhibitor targeting a wide array of mutations, including G12D, G12V, G13D, G12A, G12R, and Q61H, as well as wild-type KRAS. In preclinical studies, it effectively reduced ERK phosphorylation and inhibited KRAS-mutant tumor growth while sparing HRAS and NRAS [156].

Notably, the tricomplex RAS-ON inhibition mechanism has now been extended beyond G12D. RMC-7977 is a reversible RAS-ON inhibitor that exhibits broad-spectrum activity against both wild-type and mutant KRAS proteins, particularly those involving codon 12 substitutions (KRAS G12X) [157]. These agents may provide therapeutic options for tumors resistant to earlier KRAS inhibitors.

The identification of a druggable pocket in KRAS G13D, revealed through structural bioinformatics modeling and monoclonal antibody-guided crystallographic analysis, has broadened the therapeutic landscape. This mutation, previously difficult to target, reveals a unique open P-loop conformation that exposes the catalytic core, rendering it accessible to small-molecule inhibitors [158]. This insight is crucial for pan-KRAS and RAS-ON inhibitor development, as it expands their targetable spectrum.

Combination strategies are concurrently being investigated to improve outcomes in KRAS-mutant cancers. A key node in RAS signaling is SHP2, which facilitates full RAS activation through complexes with Grb2, Gab1, and SOS1, as characterized by biochemical signaling assays and structural mapping [116]. SHP2 inhibitors, such as SHP099 and hexachlorophene, disrupt this complex formation, thereby reducing downstream oncogenic signaling [159]. This results in decreased proliferation, increased apoptosis, and inhibition of metastasis in KRAS-driven NSCLC models [160].

Several SHP2 inhibitors, including TNO155 (NCT04000529), RMC-4630 (NCT05054725), RLY-1971 (NCT04252339), and JAB-3068 (NCT03565003), have been assessed both as monotherapies and in combination with KRAS or MAPK pathway inhibitors. However, development of agents like TNO155 and RLY-1971 was discontinued due to limited efficacy and safety concerns. Early-phase data for RMC-4630 have demonstrated preliminary activity in KRAS-mutant NSCLC, although adverse effects such as rash, fatigue, and gastrointestinal symptoms remain important considerations [161].

**Figure 3:**
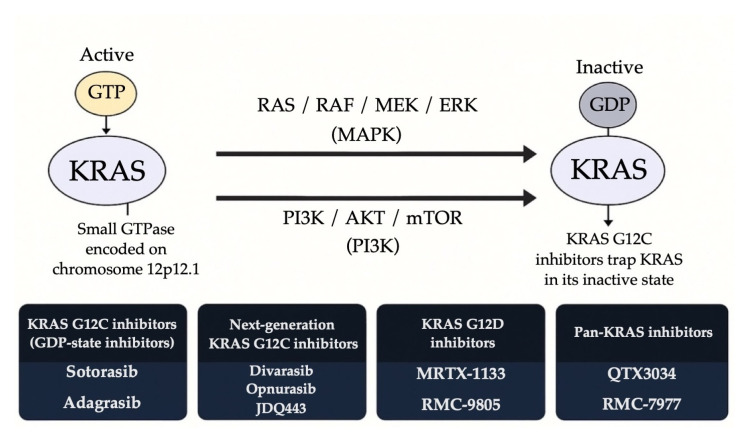
Therapeutic strategies targeting KRAS-mutant NSCLC.

Resistance mechanisms to SHP2/KRAS combination therapies, such as KRAS G12C amplification and bypass activation via the MAPK/PI3K pathway, have already been identified [136]. Despite these challenges, SHP2 inhibition remains a promising adjunct strategy. Future efforts will focus on optimizing dosing, refining patient selection, and minimizing toxicity. Figure 3 illustrates the mechanisms of KRAS activation, downstream signaling pathways, and the therapeutic strategies used to inhibit specific KRAS mutants. It highlights how KRAS mutations, particularly G12C and G12D lead to constitutive activation of MAPK and PI3K pathways, and how different classes of KRAS inhibitors target these oncogenic drivers through distinct mechanisms.

KRAS inhibitors have marked a transformative milestone in precision oncology. At present, sotorasib and adagrasib are approved and widely used as second-line treatments for advanced NSCLC harboring KRAS G12C mutations. Among the two, adagrasib appears to offer superior efficacy in patients with CNS metastases and demonstrates a more favorable safety profile when combined with immunotherapy. However, the clinical impact of these first-generation inhibitors have been limited by challenges such as short-lived responses and the emergence of resistance mechanisms. As evident by the robust pipeline of ongoing clinical trials, the field is rapidly evolving. In the coming years, it is anticipated that newer generations of KRAS-targeted therapies will emerge and potentially surpass current standards of care in both efficacy and tolerability. Table 3 provides a summary of ongoing, recently completed, and discontinued trials targeting KRAS-mutant tumors, explaining their phases, ORR, median OS, and median PFS.

**Table 3:** KRAS Targeted Therapies Clinical Trials.

Trial Name	Phase	Drugs	Median Progression Free Survival (mPFS)	Overall Response Rate (ORR)	Median Overall Survival (mOS)
CodeBreaK 100 [162]	I/II	Sotorasib [163]	6.8 months	37.1%	12.5 months
CodeBreaK 200 [164]	III	Sotorasib vs. Docetaxel [31]	5.6 months vs. 4.5 months	28.1% vs. 13.2%	10.6 months vs. 11.3 months
Kyrstal-1 [165]	I/II	Adagrasib [28]	6.5 months	43%	12.6 months
Krystal-12 [166]	III	Adagrasib vs. Docetaxel [32]	5.5 months vs. 3.8 months	32% vs. 9%	-
Divarasib trial [167]	I	Divarasib [34]	13.1 months	53%	-
Krascendo-1 [168]	III	Divarasib vs. Sotorasib or Adagrasib [169]	-	-	-
KontRASt-02 [170]	III	JDQ443 (Opunarasib) vs. Docetaxel [36]	-	-	-
KontRASt-03 [171]	I/II	JDQ443 (Opunarasib) monotherapy or with Trametinib, Ribociclib, or Cetuximab [172]	-	-	
MRTX1133 trial [173] (Terminated)	I	MRTX1133 [46]	-	-	-
HRS-4642 trial [78]	I	HRS-4642 [48]	-	-	-
MOONRAY-01 [174]	I	LY3962673 [50]	-	-	-
QTX3046 trial [175]	I	QTX3046 or with Cetuximab [51]	-	-	-
INCB161734 trial [176]	I	INCB161734 monotherapy or with Cetuximab, Retifanlimab, GEMNabP, or mFOLFIRINOX [52]	-	-	-
RMC-9805 trial [177]	I	RMC-9805 (Zoldonrasib) [54]	-	30%	-
ASP3082 trial [178]	I	ASP3082 monotherapy or with Cetuximab, Leucovorin, Oxaplatin, Fluorouracil, Irinotecan, Nanoparticle albumin-bound-paclitaxel, Gemcitabine, Docetaxel, Pembrolizumab, Cisplatin, Carboplatin, or Pemetrexed [60]	-	23.1%	-
QTX3034 trial [179]	I	QTX3034 monotherapy or with Cetuximab [93]	-	-	-
TNO155 trial [180] (Terminated)	I	TNO155 (Batoprotafib) with Spartalizumab or Ribociclib [164]	-	-	-
RMC-4630 trial [181]	II	RMC-4630 (Vociprotafib) with Sotorasib [100]	-	-	-
RLY-1971 trial [182]	I	Migoprotafib [165]	-	-	-
JAB-3068 trial [183]	I/II	JAB-3068 [94]	-	-	-

Abbreviations: ORR: Objective Response Rate; mPFS: Median Progression Free Survival; mOS: Median Overall Survival; KRAS: Kirsten rat sarcoma viral oncogene homolog; S-IIP: Switch II pocket; MEK: Mitogen-activated protein kinase; CDK: Cyclin-dependent kinase; EGFR: Epidermal growth factor receptor; GEMNabP: Gemcitabine and nab-paclitaxel; mFOLFIRINOX: Leucovorin calcium, fluorouracil, irinotecan, hydrochloride, and oxaliplatin; PD: Programmed cell death; SHP2: Src homology-2-containing protein tyrosine phosphatase 2; CSF: Colony-stimulating factor.

### 2.7. HER2

HER2, a member of the ERBB family of receptor tyrosine kinases, plays a critical role in regulating cellular growth and differentiation. Alterations involving the ERBB2 gene, located on chromosome 17, are found in approximately 1–4% of NSCLC cases [184]. These alterations can include activating mutations most commonly within the kinase domain (exons 18–21), as well as gene amplification and protein overexpression, which can be detected by mutational hotspot analysis and crystallographic modeling [184,185]. Overexpression of HER2 protein results in excessive receptor levels on tumor cells and has clinical significance in guiding the use of targeted therapies, such as trastuzumab deruxtecan (T-DXd), that directly inhibit HER2-driven oncogenic signaling [186].

Early attempts to treat HER2-altered NSCLC with pan-HER inhibitors like afatinib, dacomitinib, and neratinib showed limited success, with ORRs ranging between 0–19% and PFS of just 2.8–5.5 month [187,188]. In contrast, newer, more selective HER2-targeted TKIs have demonstrated greater efficacy. Zongertinib, a selective HER2 TKI, has shown encouraging results in early-phase trials. Preliminary data from the Beamion LUNG-1 trial (NCT04886804) revealed a 50% response rate in HER2-mutant solid tumors, with updated findings in NSCLC patients reporting an ORR of 72% and a DCR of 95.5%, along with a favorable safety profile marked by low incidences of serious gastrointestinal and hepatic toxicities [189].

BAY2927088 is another investigational agent targeting both HER2 and EGFR mutations. The phase I/II SOHO-01 trial evaluated BAY2927088 in patients with HER2-mutant NSCLC previously treated with systemic therapies. Among the 34 patients enrolled, the ORR was 70%, and responses were noted to be both rapid and durable. The median PFS was 8.1 months, and 95% of participants with detectable HER2 ctDNA at baseline experienced reductions during treatment, suggesting strong anti-tumor activity. The drug was well tolerated, with diarrhea and rash being the most frequently reported AEs [190].

The most significant breakthrough in HER2-targeted therapy for NSCLC has been the development of ADCs. T-DXd has emerged as a leading therapy, particularly in patients who have progressed on prior treatments. In the DESTINY-Lung01 trial, T-DXd achieved an ORR of 55%, with a median PFS of 8.2 months and a median OS of 17.8 months [191]. However, interstitial lung disease (ILD), reported in approximately 26% of patients, is a known and serious complication that necessitates close monitoring. The ongoing phase III DESTINY-Lung04 trial (NCT05048797) aims to assess T-DXd as a first-line therapy in comparison to standard chemotherapy plus immunotherapy [192].

Another HER2-targeting ADC, SHR-A1811, is showing promise in early-phase studies. In a phase I trial, it achieved an ORR of 41.9% and a median PFS of 8.4 months in patients with HER2-mutated NSCLC [193].

Monoclonal antibody-based regimens have also been explored. A combination of trastuzumab, pertuzumab, and docetaxel yielded an ORR of 29% and a median DOR of 11 months [15]. While these results are encouraging, monoclonal antibodies have not yet matched the clinical outcomes observed with ADCs like T-DXd.

HER2 bispecific T-cell engagers (BiTEs) represent another promising therapeutic avenue. These agents are designed to bridge HER2-expressing tumor cells and T cells to facilitate immune-mediated cytotoxicity. Preliminary clinical investigations suggest potential benefit in NSCLC, though further research is required. GBR 1302, a HER2xCD3 bispecific antibody, is terminated after reaching phase I (NCT02829372) in patients with HER2-positive solid tumors. Among 19 evaluable patients, the most common AEs were IRRs and cytokine release syndrome (CRS), particularly at doses ≥ 100 ng/kg. One patient experienced a grade 4 IRR/CRS event requiring ICU care, which resolved within 36 h. Although no objective tumor responses were observed, two patients exhibited prolonged disease stabilization lasting more than four months [194].

As HER2-targeted therapies gain a stronger foothold in the treatment landscape, it is increasingly important for clinicians to incorporate HER2 mutation and expression testing at both diagnosis and progression. This includes the utilization of liquid biopsy for the detection and monitoring of HER2 alterations, as evident by observation that detectable HER2 ctDNA reductions correlate with the anti-tumor activity of agents like BAY2927088 [190].

Despite these advancements, significant challenges remain in the management of HER2-altered NSCLC. Toxicity, especially ILD associated with ADCs, remains a key concern. Dose optimization and early detection are essential to minimize risks and improve patient safety. Moving forward, efforts will likely focus on refining therapeutic design, exploring rational drug combinations, and optimizing treatment sequencing particularly for patients with CNS involvement to improve long-term outcomes.

### 2.8. MET

The MET proto-oncogene, located on chromosome 7q21–q31, encodes a receptor tyrosine kinase that plays a key role in cellular proliferation, survival, and differentiation by activating the MAPK signaling cascade [195]. Alterations in MET account for approximately 7% of actionable mutations in NSCLC [196]. These alterations include MET exon 14 skipping mutations (METex14), gene amplifications, protein overexpression, and, less commonly, gene fusions. Among these, METex14 skipping caused by aberrant pre-mRNA splicing is the most prevalent point mutation. It results in the loss of the Y1003 tyrosine residue, leading to reduced ubiquitination and enhanced stability of the MET receptor, thereby prolonging its oncogenic activity [16,197].

METex14 alterations are detected in 3–4% of lung adenocarcinomas and in 1–2% of other NSCLC subtypes but are significantly more common (up to 20%) in pulmonary sarcomatoid carcinoma [198,199]. These mutations are generally mutually exclusive from other driver mutations such as EGFR, ALK, and KRAS [200].

MET amplification involves an increased number of MET gene copies, which activates key downstream signaling pathways, including PI3K/AKT/mTOR and RAS/RAF/MEK/ERK [197]. High-level amplification is typically defined as a MET gene copy number exceeding 10 or a MET-to-CEP7 ratio of ≥4. MET amplification is relatively uncommon, occurring in about 0.78% of lung adenocarcinomas and 1.07% of squamous cell carcinomas [201]. It can either arise de novo or as an acquired mechanism of resistance, particularly in 5–20% of patients treated with EGFR TKIs [202].

MET-directed therapies include both non-selective multikinase inhibitors (MKIs), such as crizotinib and cabozantinib, and selective MET inhibitors like capmatinib and tepotinib. The approval of capmatinib was based on results from the GEOMETRY mono-1 trial, which demonstrated an ORR of 68% (95% CI, 48–84) and a median DOR of 12.6 months in treatment-naïve patients. Previously treated patients had an ORR of 41% (95% CI, 29–53) and a DOR of 9.7 months [203]. In patients with MET amplification, responses were more pronounced in those with high copy numbers, with an ORR of 40% in untreated and 29% in previously treated individuals [203].

Tepotinib received FDA approval based on data from the VISION trial, where treatment-naïve patients with METex14-mutated NSCLC achieved an ORR of 57.3% (95% CI, 49.4–65.0) and a median DOR of 46.4 months. In previously treated patients, the ORR was 45.0% (95% CI, 36.8–53.3) with a DOR of 12.6 months [204]. Amivantamab, a bispecific antibody targeting EGFR and MET, showed a 33% ORR in METex14-mutant patients who had progressed on or declined standard therapies, as reported in the CHRYSALIS study [205].

Savolitinib is another MET TKI with demonstrated efficacy in both first line and refractory settings, including in patients with aggressive histologies such as sarcomatoid carcinoma. It is being evaluated in multiple ongoing trials for METex14-mutated NSCLC [195]. Additional non-selective MET inhibitors, including cabozantinib, merestinib, and glesatinib, are also under investigation in phase II trials (NCT01639508, NCT05613413, NCT04310007, NCT03911193).

Emerging treatment strategies include MET-targeted ADCs. REGN5093-M114, a bispecific MET-directed ADC, has demonstrated potent antitumor effects in preclinical models, particularly in METex14-mutant tumors and EGFR TKI-resistant settings [206]. It is currently being assessed in a phase I/II clinical trial for patients with MET-overexpressing advanced cancers (NCT04982224).

Telisotuzumab vedotin (Teliso-V) is another MET-targeted ADC conjugated to a monomethyl auristatin E payload. Phase I studies showed efficacy in MET-overexpressing NSCLC. The phase II LUMINOSITY trial reported an ORR of 28.6% (95% CI, 21.7–36.2) in patients with previously treated, MET-overexpressing, EGFR wild-type non-squamous NSCLC. MET overexpression was defined by IHC as >25% of tumor cells with 3+ staining and stratified into high and intermediate levels. Clinical outcomes including ORR, DOR, PFS, and OS correlated with the degree of MET expression [207].

ABBV-400, a next-generation MET-directed ADC carrying a topoisomerase I inhibitor payload, is currently being studied in early-phase clinical trials. It has shown encouraging activity and manageable toxicity profiles [208].

As new therapeutic options targeting MET alterations beyond METex14 emerge, it is increasingly important for clinicians to assess MET status including overexpression and amplification especially in patients progressing on prior lines of therapy, to guide optimal MET-targeted treatment selection.

### 2.9. RET

The RET proto-oncogene, located on chromosome 10q11.2, encodes a receptor tyrosine kinase involved in regulating cellular growth, differentiation, and survival. RET mutations have long been associated with multiple endocrine neoplasia type 2 (MEN2) and thyroid cancers [209]. In 2011, RET fusions were identified as oncogenic drivers in NSCLC, expanding its relevance beyond endocrine malignancies [210]. Among the various fusion partners discovered, the KIF5B-RET fusion is the most frequently observed [211]. These fusion proteins lead to persistent activation of RET kinase signaling and promote tumorigenesis via downstream pathways such as MAPK and PI3K/AKT [17].

RET fusions occur in approximately 1–2% of NSCLC cases, predominantly among patients with adenocarcinoma histology, younger age, and limited smoking history [212,213]. Initial treatment efforts with MKIs—such as cabozantinib, vandetanib, and lenvatinib—showed limited efficacy and were associated with significant off-target toxicities, making them suboptimal options for RET-rearranged NSCLC [18,214,215,216].

The treatment landscape changed significantly with the development of selective RET inhibitors. Selpercatinib, a highly selective RET kinase inhibitor capable of crossing the blood–brain barrier, demonstrated substantial clinical benefit. In the LIBRETTO-001 trial, it achieved an ORR of 64% (95% CI, 54–73) and a median DOR of 17.5 months (95% CI, 12.0–NE) in patients with advanced RET-fusion NSCLC. Intracranial activity was particularly notable, with a 91% response rate among patients with measurable CNS metastases. Treatment was generally well tolerated, with hypertension, elevated liver enzymes, hyponatremia, and lymphopenia being the most common grade ≥3 AEs. The discontinuation rate due to toxicity was low, at just 2% [217,218].

The phase III LIBRETTO-431 trial confirmed selpercatinib′s superiority over standard chemotherapy or chemo-immunotherapy combinations. The trial demonstrated a median PFS of 24.8 months with selpercatinib versus 11.2 months in the control arm (HR 0.31–0.70; *p* < 0.001), and an ORR of 84% compared to 65% with standard treatment [219].

Pralsetinib is another selective RET inhibitor evaluated in the ARROW phase I/II trial, which included both treatment-naïve and previously treated RET-rearranged NSCLC patients. ORRs were 72% (95% CI, 60–82) and 59% (95% CI, 50–67) in treatment-naïve and pretreated groups, respectively, leading to its accelerated FDA approval in 2020 [220]. Updated results show an ORR of 72% and a median PFS of 13.0 months in treatment-naïve patients, while pretreated patients demonstrated an ORR of 59% and PFS of 16.5 months [220]. Common side effects include neutropenia, hypertension, elevated creatine phosphokinase, and lymphopenia. The drug maintains a favorable safety profile [218]. The ongoing phase III AcceleRET Lung trial (NCT04222972) is comparing pralsetinib to the current standard of care in the first-line setting [221].

A recent case report suggests that pralsetinib may exhibit off-target activity in tumors harboring ALK rearrangements. In a patient with ALK fusion-positive NSCLC who had progressed on multiple lines of ALK-directed therapy, pralsetinib led to a durable clinical and radiographic response [222]. This finding highlights a promising future direction in structure-based drug repositioning, particularly in treatment refractory NSCLC. 

Resistance to selpercatinib and pralsetinib can develop through both RET-dependent mechanisms, such as the solvent-front G810X mutation, and RET-independent alterations, including MET and KRAS amplifications. These were identified through post-progression tumor sequencing and structural modeling, which revealed impaired binding at the kinase active site [223,224]. These solvent-front alterations have guided the design of next-generation RET inhibitors with modified hinge and solvent-channel engagement geometries, as such mutations can hinder drug binding and contribute to disease progression.

Several next-generation RET inhibitors are under development to address resistance. Although TPX-0046, a RET/SRC dual inhibitor, showed early preclinical promise, its clinical development was discontinued due to toxicity concerns [225]. Novel compounds such as HSN608, HSL476, and HSL468 part of the ALKynyl nicotinamide class have demonstrated potent preclinical activity against RET resistance mutations, including solvent-front (G810X) and gatekeeper (V804M) variants [226].

Although RET fusions are relatively rare, routine testing is essential, especially in younger, non-smoking patients without other targetable mutations. RNA-based next-generation sequencing along with DNA-based platforms for detecting RET rearrangements is preferred over DNA-based testing as it offers superior sensitivity [227].

### 2.10. FGFR

The FGFR signaling pathway plays a critical role in regulating cell proliferation, survival, differentiation, and angiogenesis [19]. Aberrations in FGFR can drive oncogenesis through gene amplifications, activating mutations, or chromosomal translocations [228]. Additionally, FGFR pathway activation has been identified as a compensatory resistance mechanism in tumors treated with EGFR or KRAS-targeted therapies [229,230].

FGFR1–3 fusions represent a unique molecular subset of NSCLC, more frequently observed in patients with a smoking history [231]. FGFR mutations are most commonly found in squamous cell carcinoma (6.8%), whereas they are less common in other NSCLC subtypes (approximately 1.3%) [232,233,234].

Currently, no FGFR-targeted therapies are approved specifically for NSCLC. However, several agents have been approved in other tumor types. Pemigatinib and futibatinib are approved for advanced cholangiocarcinoma with FGFR2 fusions, while erdafitinib is approved for metastatic urothelial carcinoma with FGFR2 or FGFR3 alterations [235,236].

Off-label use of these agents in NSCLC has shown potential. In one reported case, a patient with advanced squamous cell lung cancer harboring an FGFR3-TACC3 fusion—identified via RNA sequencing and fusion transcript analysis—experienced sustained clinical benefit and disease control for 11 months while on erdafitinib therapy [237]. These observations underscore the therapeutic potential of FGFR inhibition in selected NSCLC patients. Practically, RNA-based fusion calling increases sensitivity in small lung biopsies and liquid biopsies, facilitating the identification of rare events amenable to targeted therapy.

Ongoing studies are further exploring the role of FGFR inhibitors in NSCLC. LOXO-435, a selective FGFR3 inhibitor, is currently being evaluated in a phase I trial enrolling patients with advanced FGFR3-altered solid tumors, including NSCLC (NCT05614739). The results of this study may provide critical insight into the future of FGFR-targeted therapy in lung cancer.

### 2.11. TROP-2

TROP-2 is a transmembrane glycoprotein and a member of the epithelial cell adhesion molecule (EpCAM) family [238]. Originally identified in trophoblasts, TROP-2 was later found to be significantly overexpressed in several epithelial malignancies, with minimal expression in normal tissues [238,239]. In 2008, TROP-2 was classified as an oncogene, as its suppression reduced tumor invasiveness, highlighting its therapeutic potential [240]. In NSCLC, TROP-2 expression is observed in approximately 75% of squamous cell carcinomas, 65% of adenocarcinomas, and 18% of high-grade neuroendocrine tumors [241]. Notably, higher TROP-2 levels in lung adenocarcinomas have been associated with increased cancer-specific mortality [241].

Functionally, TROP-2 promotes oncogenesis through activation of several signaling cascades, including PTEN/PI3K/Akt, MAPK/ERK, JAK/STAT, ErbB, TGF-β, and WNT/β-catenin pathways, thereby enhancing tumor cell survival and proliferation [242]. Because of its high tumor-specific expression and correlation with poor outcomes, TROP-2 has emerged as an attractive therapeutic target [243].

**Figure 4:**
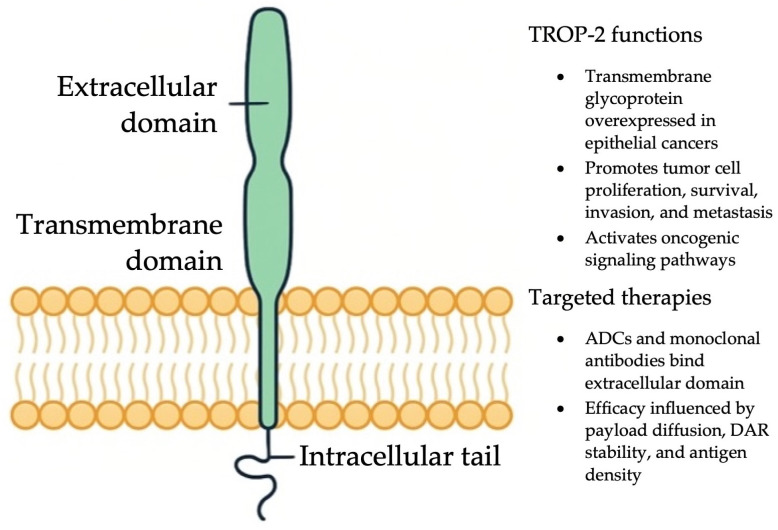
Mechanisms of action of ADCs and monoclonal antibodies targeting TROP-2 in cancer cells.

One of the most advanced TROP-2–targeted therapies is sacituzumab govitecan (SG), an ADC consisting of a humanized anti TROP-2 monoclonal antibody linked to SN-38, the active metabolite of irinotecan, via a cleavable linker [244]. Figure 4 provides an overview of TROP-2 structure and therapeutic targeting, illustrating how SG binds to overexpressed TROP-2 on the tumor surface and delivers its cytotoxic payload intracellularly. With a high drug-to-antibody ratio (DAR) of 7.6:1, SG can deliver potent cytotoxic effects even in tumors with modest antigen expression [244]. Early-phase trials in advanced solid tumors not selected for TROP-2 status showed encouraging efficacy and manageable toxicity [245,246]. SG received FDA approval for metastatic triple-negative breast cancer following results from the IMMU-132-01 trial, which demonstrated an ORR of 33.3% and a median DOR of 7.7 months [247].

In a subset of 54 heavily pretreated NSCLC patients included in an expansion cohort, SG achieved an ORR of 17% (intention-to-treat population) and 19% among evaluable patients, with a median DOR of 6.0 months and a PFS of 5.2 months [248].

More recently, the phase III EVOKE-01 trial compared SG to docetaxel in previously treated metastatic NSCLC. Although the primary endpoint of OS was not statistically met, SG demonstrated a numerical OS improvement (median OS 11.1 vs. 9.8 months; HR 0.84; 95% CI, 0.68–1.04) [249]. A notable benefit was observed in patients who had not responded to their most recent immunotherapy-containing regimen, with a median OS of 11.8 vs. 8.3 months (HR 0.75; 95% CI, 0.58–0.97) [249].

Several trials are evaluating SG in combination regimens. The ongoing EVOKE-02 trial (NCT05186974) is assessing SG with pembrolizumab ± platinum chemotherapy in treatment-naïve metastatic NSCLC with PD-L1 ≥ 50% and no actionable alterations. Early safety data from cohort A (SG + pembrolizumab) noted grade ≥3 treatment-related adverse events (TRAEs) including neutropenia, diarrhea, and respiratory failure [20]. Response data are pending. In parallel, EVOKE-03 (NCT05609968) is comparing SG plus pembrolizumab vs. pembrolizumab monotherapy in advanced NSCLC.

In parallel with antibody-drug conjugates and anti-PD-1 strategies, dual immune checkpoint blockade with nivolumab plus ipilimumab has shown durable benefit. The Checkmate 227 trial (NCT02477826) reported a 5-year survival rate comparing first-line nivolumab + ipilimumab compared to platinum-doublet chemotherapy in metastatic NSCLC. In patients with PD-L1 ≥ 1%, the 5-year OS was 24% with the combination and 14% with chemotherapy; the corresponding rates were 19% versus 7% in patients with PD-L1 < 1%. Median DOR was also considerably longer, with 24.5 months versus 6.7 months for PD-L1 ≥ 1% and 19.4 versus 4.8 months for PD-L1 < 1%. Interestingly, 64–66% of 5-year survivors were off systemic therapy for ≥3 years, with preserved quality of life. These results validate dual checkpoint blockade as a chemotherapy-free, long-lasting option regardless of PD-L1 status [250].

Another TROP-2–targeting ADC in development is datopotamab deruxtecan (Dato-DXd). The phase I TROPION-PanTumor01 study showed promising activity in heavily pretreated patients [251,252,253], prompting further investigation in NSCLC. In the phase III TROPION-Lung01trial, Dato-DXd demonstrated a statistically significant improvement in PFS compared to docetaxel (4.4 vs. 3.7 months; HR 0.75; 95% CI, 0.62–0.91; *p* = 0.004), particularly in non-squamous NSCLC (5.5 vs. 3.6 months) [254]. However, no OS benefit was observed (7.6 vs. 9.4 months), leading to withdrawal of the biologics license application for second-line use.

Subsequent biomarker analysis identified that TROP-2 expression measured by normalized membrane ratio (NMR) was predictive of benefit. In patients with TROP-2 QCS-NMR positivity (≤0.56), Dato-DXd was associated with improved PFS compared to docetaxel (6.9 vs. 4.1 months; *p* = 0.0063) [163].

Ongoing studies are now exploring Dato-DXd in earlier lines of therapy and in combination regimens. The TROPION-Lung02 trial includes previously treated and treatment-naïve patients receiving Dato-DXd with pembrolizumab ± chemotherapy [255]. Most common side effects include nausea, stomatitis, neutropenia, elevated amylase, and ILD. Further data are awaited.

TIGIT has been an attractive target for immune checkpoint therapy. Combination of atezolizumab (anti-PD-L1) with tiragolumab (anti-TIGIT) in the CITYSCAPE trial provided substantially improved response rate and progression-free survival compared to atezolizumab alone in previously untreated PD-L1-positive NSCLC patients [256]. Recent studies have identified tumor-associated macrophage activity and regulatory T-cell dynamics as potential response biomarkers, hence incorporating immune contexture biomarkers in decision making. 

Concurrently, LAG-3 and TIM-3 are next-generation immune checkpoints that are being investigated for NSCLC. Both receptors are commonly co-expressed with PD-1 and play a role in T-cell exhaustion and tumor immune evasion. LAG-3 binds MHC class II and suppresses CD4+ and CD8+ T-cell function and enhances Treg-mediated immunosuppression, while TIM-3 binds ligands such as galectin-9 and phosphatidylserine to promote immune tolerance and inhibit Th1 responses. Preclinical and early phase studies have demonstrated that co-blockade of these pathways, particularly when combined with anti-PD-1/PD-L1 therapies, can synergistically restore anti-tumor immunity. There are multiple trials testing dual and triple checkpoint inhibition strategies with LAG-3 and TIM-3 in PD-L1-specific NSCLC [257].

Other notable trials include:

- TROPION-Lung04 (NCT04612751): A phase 1b study evaluating Dato-DXd with various immunotherapies (durvalumab, anti–PD-1/TIGIT, anti–PD-1/CTLA-4) in newly diagnosed NSCLC patients without targetable mutations, stratified by PD-L1 expression [258].
- TROPION-Lung05 (NCT04484142): A phase II study assessing Dato-DXd in patients with actionable genomic alterations who progressed on prior targeted or platinum therapies, with an ORR of 35.8% [169].
- Ongoing trials are exploring Dato-DXd in combination with osimertinib (NCT06417814, NCT06350097), rilvegostomig (NCT06357533), and pembrolizumab (NCT05555732, NCT05215340) [172,259,260,261,262].

### 2.12. Emerging Therapies

The success of mRNA technology in infectious disease has driven interest in its extension into oncology. In NSCLC, mRNA vaccines provide a platform for inducing tumor-specific immune responses through encoding neoantigens or tumor-associated antigens. Vaccines such as CV9201 have shown immunogenicity in early-phase studies, and second-generation vaccines like BNT116 and V940 (mRNA-4157) are under evaluation in combination with checkpoint inhibitors in resectable and advanced NSCLC. They allow for immediate, personalized design and can code for multiple epitopes to stimulate CD4+ and CD8+ T cells with widespread activity. Despite this, there remain concerns of mRNA stability, tumor-specific targeting, and immune evasion within the tumor microenvironment. Upcoming trials will determine their therapeutic value in the NSCLC treatment protocol [263,264]. Table 4 provides a concise overview of the eight key clinical trials evaluating mRNA vaccines in NSCLC, detailing targets, delivery platforms, phases, and status [263].

**Table 4:** Emerging Therapies Clinical Trials.

Clinical Trial Number	Antigen Targets	Delivery Platforms	Phase	Status
NCT00004604 [265]	CEA	Dendritic cells	I	Completed
NCT00923312 [266]	NY-ESO-1, MAGE-C1/C2, survivin, and trophoblast glycoprotein	Protamine	I/II	Completed
NCT01915524 [267]	MUC1, survivin, NY-ESO-1, 5T4, MAGE-C2, and MAGE-C1	Protamine	I	Terminated
NCT03164772 [268]	MUC1, survivin, NY-ESO-1, 5T4, MAGE-C2, and MAGE-C1	N/A	I/II	Completed
NCT02688686 [269]	SOCS 1, MUC1, and Survivin	DC, CIK	I/II	Unknown
NCT03948763 [270]	KRAS	LNP	I	Terminated
NCT05202561 [271]	KRAS	N/A	I	Recruiting
NCT03908671 [272]	N/A	N/A	N/A	Recruiting

Abbreviations: DC: dendritic cells; CIK: cytokine-induced killer; LNP: lipid nanoparticle; CEA: carcinoembryonic antigen; NY-ESO-1: New York esophageal squamous cell carcinoma-1; MAGE: melanoma antigen family; MUC1: mucin1; SOCS: suppressor of cytokine signaling; KRAS: Kirsten rat sarcoma viral oncogene homolog.

Apart from vaccines, there are also engineered cell therapies being studied in NSCLC, with a focus on both chimeric antigen receptor (CAR)-T cells and T-cell receptor (TCR)-engineered T cells. Several CAR-T cell therapies are targeting EGFR, MUC1, and mesothelin (MSLN), and other tumor antigens and are being tested in early-phase clinical trials. CAR-T cell designs range from first generation CD3 based structures to fifth generation armored CARs capable of cytokine secretion, being structured to address the challenges in solid tumor microenvironments. These include poor T-cell penetration, immunosuppressive signaling, and antigen heterogeneity. Engineered TCR T-cells, which recognize intracellular peptides presented on MHC molecules, have the potential to target antigens beyond surface epitopes. Preclinical and initial clinical trials are assessing both cell types in NSCLC [273].

## 3. Conclusions

The therapeutic landscape of NSCLC is rapidly evolving, driven by the emergence of molecularly targeted treatments against defined oncogenic alterations, as illustrated in Figure 2. This review highlights significant strides in the development of precision therapies directed at key mutations and alterations in KRAS, EGFR, HER2, MET, ALK, RET, BRAF V600E, NTRK, ROS1, TROP-2, and FGFR, offering renewed hope for patients who historically had limited treatment options. The success of next-generation TKIs and ADCs underscore a paradigm shift toward more personalized approaches in lung cancer care. These advancements also emphasize the critical role of comprehensive molecular profiling at both diagnosis and disease progression to identify actionable targets and guide therapeutic decision-making. As the field progresses, ongoing research into resistance mechanisms, rational drug combinations, and biomarker-driven strategies will be pivotal in optimizing treatment efficacy. Ultimately, the future of NSCLC management lies in precision oncology, where therapies are tailored to the individual genetic makeup of each tumor, aiming to improve survival outcomes and quality of life for cancer patients.

## Figures and Tables

**Table 1:** NSCLC targets, therapeutic classes, and mechanisms of action.

Target	Common Alterations	Therapeutic Classes and Representative Agents	Mechanism of Action
EGFR [9]	Ex19del, L858R, T790M, Exon20ins; uncommon (G719x, L861Q, S768I, etc.)	TKIs (geftinib, erlotinib, afatinib, dacomitinib, osimertinib, furmonertinib, sunvozertinib); EGFR/MET bispecific (amivantamab ± lazertinib); HER3-ADC (patritumab deruxtecan)	TKIs: inhibit EGFR kinase domain; Amivantamab: blocks EGFR and MET receptors; HER3-ADC: internalization of ADC complex via HER3 and delivery
ALK [10]	Fusions (e.g., EML4-ALK)	TKIs (crizotinib, alectinib, brigatinib, ceritinib, lorlatinib; emerging deulorlatinib, NVL-655)	Inhibit ALK kinase (receptor tyrosine kinase fusion) preventing downstream MAPK/PI3K signaling
ROS1 [11]	Fusions	TKIs (crizotinib, enterctinib, lorlatinib, repotrectinib, taletrectinib)	Inhibit ROS1 fusion kinase to block downstream signaling (often CNS-active)
NTRK [12]	Fusions	TRK inhibitors (larotrectinib, entrectinib)	Inhibit TRK kinase domain, shutting downstream cascades
BRAF V600E [13]	V600E mutation	BRAF + MEK inhibitors (dabrafenib + trametinib)	Inhibit mutant BRAF kinase and downstream MEK to suppress MAPK signaling pathway
KRAS [14]	G12C, G12D, G12V	G12C inhibitors (sotorasib, adagrasib, divarasib, opunarasib); G12D inhibitors (MRTX-1133, HRS-4642); RAS-ON inhibitors (RMC-9805); degraders (ASP3082); SHP2 inhibitors (RMC-4630)	Direct RAS inhibition (nucleotide-state or switch-II pocket); degraders remove KRAS protein; SHP2 inhibitors block upstream RAS activation
HER2 (ERBB2) [15]	Kinase domain mutations (ex18–21, specifically exon20ins); amplification/overexpression	ADCs (trastuzumab deruxtecan; SHR-A1811); TKIs (zongertinib, BAY2927088; afatinib/dacomitinib)	ADCs: HER2 bindings leading to internalization and degradation within the cell; TKIs: inhibit HER2 kinase signaling
MET [16]	Exon 14 skipping; amplification; overexpression	Selective TKIs (capmatinib, tepotinib, savolitinib); EGFR/MET bispecific (amivantamab); ADCs (REGN5093-M114, telisotuzumab vedotin, ABBV-400)	TKIs: inhibit MET kinase; bispecific: block ligand/receptor and induce internalization; ADCs: receptor-mediated internalization leading to cytotoxicity
RET [17]	Fusions (e.g., KIF5B-RET)	Selective TKIs (selpercatinib, pralsetinib)	Inhibit RET fusion kinase to block downstream signaling
FGFR [18]	Fusions (FGFR2/3), mutations, amplifications (more in squamous)	FGFR inhibitors (erdafitinib, futibatinib, pemigatinib—off-label/selected cases; LOXO-435 investigational)	Inhibit FGFR kinase to block downstream signaling
TROP2 [19]	Overexpression	ADCs (sacituzumab govitecan, datopotamab deruxtecan)	Antibody binding → internalization → SN-38 or DXd payload-mediated damage
Immune checkpoints [20]	PD-L1 expression; TMB/MSI	PD-1/PD-L1 (pembrolizumab, nivolumab, atezolizumab); CTLA-4 (ipilimumab); emerging TIGIT/LAG-3/TIM-3	Block inhibitory receptor-ligand interactions at the immune synapse to restore T-cell signaling

Abbreviations: EGFR: Epidermal Growth Factor Receptor; TKI: Tyrosine Kinase Inhibitor; MET: Mesenchymal-Epithelial Transition; HER: Human Epidermal Growth Factor Receptor; ADC: Antibody-drug conjugate; ALK: Anaplastic lymphoma kinase; EML4: Echinoderm microtubule-associated protein-like 4; MAPK: Mitogen-activated protein kinase; PI3K: Phosphoinositide 3-kinase; NTRK: Neurotrophic receptor tyrosine kinase; TRK: Tropomyosin receptor kinase; BRAF: B-rapidly accelerated fibrosarcoma gene; MEK: Mitogen-activated extracellular kinase; RAS: Rat sarcoma viral oncogene homolog; RET: Rearranged during Transfection; FGFR: Fibroblast growth factor receptor; TROP: Trophoblast cell surface antigen.

**Table 2:** EGFR Targeted Therapies Clinical Trials.

Trial Name	Phase	Drugs	Median Progression Free Survival (mPFS)	Overall Response Rate (ORR)
LAURA [63]	III	Osimertinib vs. placebo [64]	39.1 months vs. 5.6 months	-
FLAURA 2 [65]	III	Osimertinib monotherapy or combined with platinum-based chemotherapy (pemetrexed + either cisplatin or carboplatin) [66]	25.5 months vs. 16.7 months	83% vs. 76%
MARIPOSA [67]	III	Osimertinib vs. Amivantamab and Lazertinib [68]	16.6 months vs. 23.7 months	85% vs. 86%
MARIPOSA II [69]	III	Amivantamab + chemotherapy (carboplatin + pemetrexed) vs. Amivantamab + Lazertinib + chemotherapy (with protocol modification to start Lazertinib after carboplatin) vs. Chemotherapy alone (carboplatin + pemetrexed) [70]	6.3 months vs. 8.3 months vs. 4.2 months Median intracranial PFS 12.5 months vs. 12.8 months vs. 8.3 months	64% vs. 63% vs. 36%
PALOMA 3 [71]	III	Subcutaneous Amivantamab + Lazertinib vs. IV regimen [72]	6.1 months vs. 4.3 months	30% vs. 33%
PAPILLON [73]	III	Amivantamab + chemotherapy vs. chemotherapy (carboplatin + pemetrexed) [74]	11.4 months vs. 6.7 months	73% vs. 47%
WU-KONG 1 [75]	I/II	DZD9008 (Sunvozertinib) [76]	-	54.3%
WU-KONG 28 [77]	III	DZD9008 (Sunvozertinib) vs. Platinum-based doublet chemotherapy (pemetrexed + carboplatin) [78]	-	-
FURLONG [79]	III	Furmonertinib vs. Geftinib [80]	20.8 months vs. 11.1 months	-
FURVENT/FURMO-004 [81]	III	Furmonertinib [82]	-	78.6%
HERTHENA-Lung 01 [83]	II	Patritumab deruxtecan [13]	5.5 months	29.8%
HERTHENA-Lung 02 [84]	III	Patritumab deruxtecan monotherapy vs. with chemotherapy [85]	5.5 months	29.8%
Beamion-Lung 01 [86]	I	Zongertinib [87]	12.4 months in cohort 1	66.7% at 120 mg and 72–78% at 120 mg and 240 mg
ACHILLES/TORG 1834 [88]	III	Afatinib vs. chemotherapy (cisplatin or carboplatin + pemetrexed) [89]	10.6 months vs. 5.7 months	Afatinib 61.7%, G719X 55.8%, L861Q 72.7%, S768I 60%
ORIC-114 [90]	I/II	ORIC-114 [91]	-	-

Abbreviations: EGFR: Epidermal Growth Factor Receptor; TKI: Tyrosine Kinase Inhibitor; MET: Mesenchymal-Epithelial Transition; IV: Intravenous; HER: Human Epidermal Growth Factor Receptor.

## Data Availability

No new data were created or analyzed in this study. Data sharing is not applicable to this article.
